# A Study of the Prevalence of Anemia in Children With Severe Acute Malnutrition at a Pediatric Tertiary Care Hospital in South India

**DOI:** 10.7759/cureus.67657

**Published:** 2024-08-24

**Authors:** Womesh Chandra Venigalla, C Nirmala, Cherukuri Harshita, Sritej Reddy Meghi

**Affiliations:** 1 Pediatrics, Osmania Medical College, Hyderabad, IND; 2 Pediatrics, Niloufer Hospital, Hyderabad, IND; 3 Pediatrics, Apollo Hospitals, Hyderabad, IND

**Keywords:** folate deficiency, vit b12 deficiency, under 5 age group, iron-deficiency, nutritional anemia, severe acute malnutrition (sam)

## Abstract

Introduction

In developing countries such as India, severe acute malnutrition (SAM) has been a cause for great concern in the pediatric population. SAM is associated with significant morbidity and mortality in children less than 60 months of age and leaves them vulnerable to diseases due to a decrease in immunological response. Children with SAM are prone to infections, and due to nutritional deficiency, many have anemia which may be a direct or indirect cause of morbidity and mortality. They are affected by frequent respiratory and gastrointestinal infections.

Methodology

A cross-sectional study was conducted for a period of two months, from December 1, 2023, to January 31, 2024, in children with SAM aged less than 60 months. A detailed history and demographic profile were taken and recorded in a predesigned proforma. Anthropometric measurements of the study subjects were recorded, and lab investigations included complete blood picture, serum iron, serum ferritin, serum folate, and serum vitamin B12 levels. The prevalence and severity of anemia were determined by assessing the hemoglobin levels. The data collected was analyzed in Excel sheets (Microsoft Corporation, Redmond, Washington, United States) and the results were depicted in the form of graphs.

Results

A total of 300 children were included in the study of which 22 children were aged less than six months and 278 children were in the age group of 6-60 months. The overall gender distribution was 124 (41.4%) males and 176 (58.6%) females. In the age group of <6 months, of the 22 children, six (27.27%) were females while 16 (72.72%) were male. In the age group of 6-60 months, of the 278 children, 170 (61%) were females while 108 (39%) were males. Of the total 300 children, 232 (77.3%) were found to be anemic, of which 54 (23.2%) had mild anemia, 162 (69.8%) had moderate anemia, and 16 (6.89%) had severe anemia. Low serum iron levels were detected in 134 (44.6%) with iron deficiency being more common in females; below-normal ferritin levels were seen in 153 (51%) cases. Folate levels were found to be deficient in 97 (32.3%) children while vitamin B12 levels were deficient in 186 (62%).

Conclusion

Anemia is a common occurrence in children with SAM. Prevention of anemia starts from the womb by improvement of maternal nutrition and iron, and folic acid supplementation during pregnancy. Exclusive breastfeeding up to six months of age and further continuation of breastfeeding coupled with initiation of home-available complementary feeding from the age of six months onwards go a long way in maintaining healthy nutrition status in children in the vulnerable age group of less than 60 months. Healthcare professionals should utilize the well-baby and well-child visits to educate the parents and primary caretakers regarding the feeding practices to prevent, detect, and treat anemia, which will help reduce the morbidity and mortality in children with SAM.

## Introduction

In developing countries such as India, Severe Acute Malnutrition (SAM) leading to significant pediatric morbidity and mortality has been of great concern. Although malnutrition is extremely rampant in India, it is often overlooked [1].

According to the National Family Health Survey (NFHS)-3, 2005-06, 6.4% of children below the age of 60 months, i.e., nearly 8.1 million, were estimated to suffer from SAM [2,3]. The prevalence of SAM in children had risen to 7.4% according to the NFHS-4, 2015-16 [4] and further increased to 7.7% according to the NFHS-5, 2019-20 [5], which indicates increasing prevalence over the years. This can be further affected by the COVID-19 pandemic, which deprived many of job opportunities and pushed millions into poverty, reducing the incomes of many and disproportionately affecting the economically disadvantaged, leading to malnutrition and food insecurities [6].

SAM is defined as a severely low weight-for-height or weight-for-length (Z score < -3 SD, according to the median WHO child growth standards) and/or a mid-upper arm circumference (MUAC) of less than 11.5 cm, and/or by the presence of bilateral pedal edema [7]. Inadequate breastfeeding, delayed commencement of complementary feeding, feeding diluted sub-nutritious foods, recurrent gastrointestinal and respiratory tract infections, ignorance, and poverty constitute some of the factors that may lead to SAM.

SAM is associated with significant morbidity and mortality in children less than 60 months of age and leaves them vulnerable to diseases due to a decrease in immunological response. These children are prone to infections, and due to nutritional deficiency, over 80% have anemia which may be a direct or indirect cause of morbidity and mortality. They are often affected with frequent respiratory and gastrointestinal infections which further aggravates the calorie-protein deficit. Children with SAM are nine times more prone to death than well-nourished children [8,9].

The primary objective of this study was to determine the prevalence of anemia in children < 60 months of age with SAM. The secondary objective of this study was to assess the severity of anemia, serum iron, ferritin, folic acid, and vitamin B12 levels in children with SAM and provide appropriate treatment.

## Materials and methods

This was an observational cross-sectional study conducted at Niloufer Hospital, Lakdikapul, Hyderabad, for a duration of two months, from December 1, 2023, to January 31, 2024, among children with SAM aged less than 60 months of age. The study protocol was approved by the Institutional Ethical Committee of Osmania Medical College (approval number: IEC-BHR/OMC/M. NO(04)/P-45).

The study included 300 children with SAM who attended the outpatient services and children who were admitted to the hospital and satisfied the SAM criteria were included in the study. In addition, children with SAM admitted to the nutritional rehabilitation centre of the hospital were included in the study.

Inclusion and exclusion criteria

All children in the age group of less than 60 months with SAM whose parents gave consent were included in the study.Children with SAM who had structural disorders of cardiac, renal, and other systems were excluded from the study. Children whose parents had not given consent were excluded from the study. Children who were already started on treatment with haematinics or received blood transfusions were excluded from the study. Neonates were excluded from the study.

Data collection procedures

After obtaining written informed consent from the legal guardian, a detailed history and demographic profile were taken and recorded in a predesigned proforma. The anthropometric data was used to assess the nutritional status and identify children with SAM. The criteria for SAM for children between six months to 60 months are: (i) very low weight‑for-height/length (Z-score below -3 SD of the median WHO child growth standards), (ii) a mid-upper arm circumference < 11.5 cm, or (iii) the presence of bilateral pedal edema [7]. The criteria for SAM for children less than six months are: (i) visible severe wasting, (ii) weight-for-length Z scores less than -3 SD, and (iii) bilateral pedal edema [9].

Anemia in children was defined as blood hemoglobin <11g/dl and classified into mild (10-10.9g/dl), moderate (7-9.9g/dl), and severe anemia (<7g/dl). The cutoff levels for serum iron, ferritin, folate, and vitamin B12 were taken as 50 mcg/dl, 50 ng/ml, 5ng/ml, and 350 ng/L respectively. The mid-upper arm circumference was measured by using a color-coded tape. The weight of the child was taken with minimal clothing using a standardized digital weighing scale, the length was recorded using an infantometer, and the height by a stadiometer. The weight‑for-length Z-score (WLZ) and weight-for-length Z-score (WHZ) were calculated based on the WHO growth reference charts from the obtained information. At enrollment, 3 ml of the venous blood sample was collected from the dorsum of the hand with a syringe out of which 1 ml of the blood sample was collected in a purple top vacutainer with ethylenediamine tetraacetic acid (EDTA) as the anticoagulant and was sent to the pathology department for a complete blood picture (CBP). Hemoglobin levels were estimated by a five-part hematology analyzer, SYSMEX XN 1000. The machine was regularly subjected to internal and external control validation. The remaining 2 ml of venous blood was collected in a red vacutainer (no anticoagulant) and sent to the biochemistry laboratory for estimation of serum iron, serum ferritin, serum B12, and serum folate levels on a fully automated Siemens chemiluminescence autoanalyzer.

Statistical analysis

The data was collected in Google Forms (Google LLC, Mountain View, California, United States) during the study period. The data collected was analyzed in Excel sheets (Microsoft Corporation, Redmond, Washington, United States) and the results were depicted in the form of graphs and tables.

## Results

**Figure 1:**
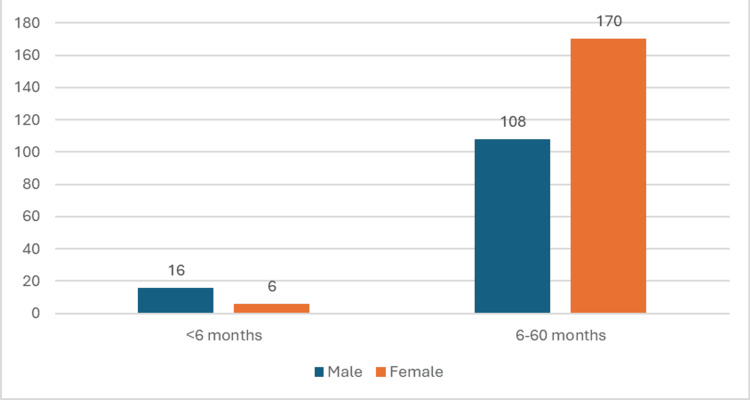
Gender and age distribution of the study population (N=300) The data has been represented as n

A sample population of 300 children aged < 60 months was included in this study which comprised 22 (7.4%) children aged <6 months and 278 (92.6%) children aged 6-60 months. The overall gender distribution was 124 (41.4%) males and 176 (58.6%) females as depicted in Figure 1 and Table 1. Females were more affected in the age group of 6-60 months, while males were more affected in the age group of <6 months. Of the 22 children in the age group of <6 months, six (27.28%) were females, while 16 (72.72%) were male. Of the 278 children in the age group of 6-60 months, 170 (61%) were females while 108 (39%) were males.

**Table 1:** Gender distribution of the study population (N=300) The data has been represented as n and %

Gender	Number	Percentage
Male	124	41.4%
Female	176	58.6%

**Figure 2:**
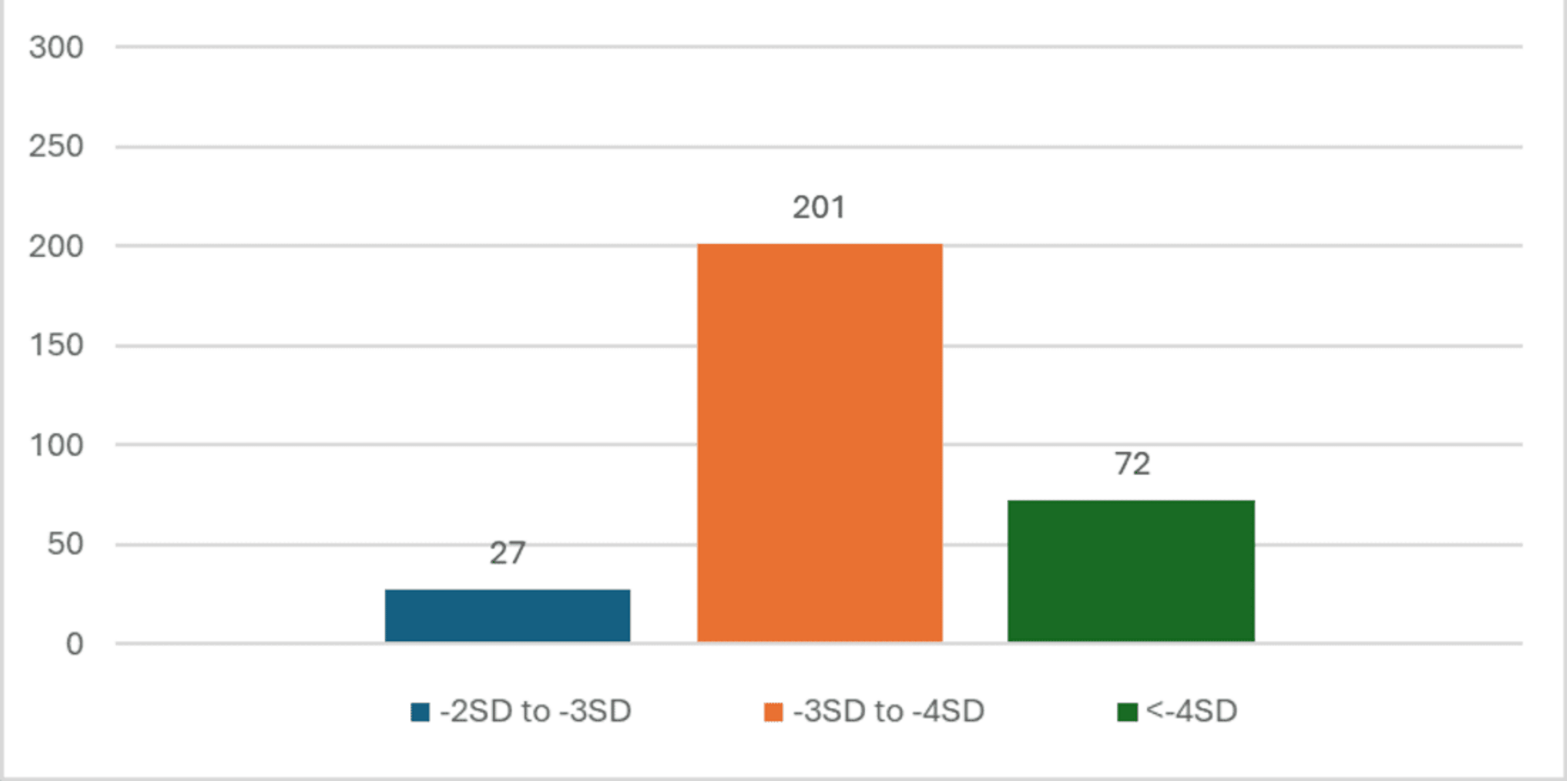
Weight for length/height Z-scores in study subjects (N=300) The data has been represented as n

The study found that among the 300 SAM children, MUAC was less than 11.5 cms in 191 (63.6%) children and the weight for height/length Z-score was -2 SD to -3 SD in 27 (9%) cases, -3 SD to -4 SD in 201 (67%) cases, and <-4 SD in 72 (24%) cases as depicted in Figure 2. Of the total 300 children, seven (2.3%) had edematous malnutrition.

**Figure 3:**
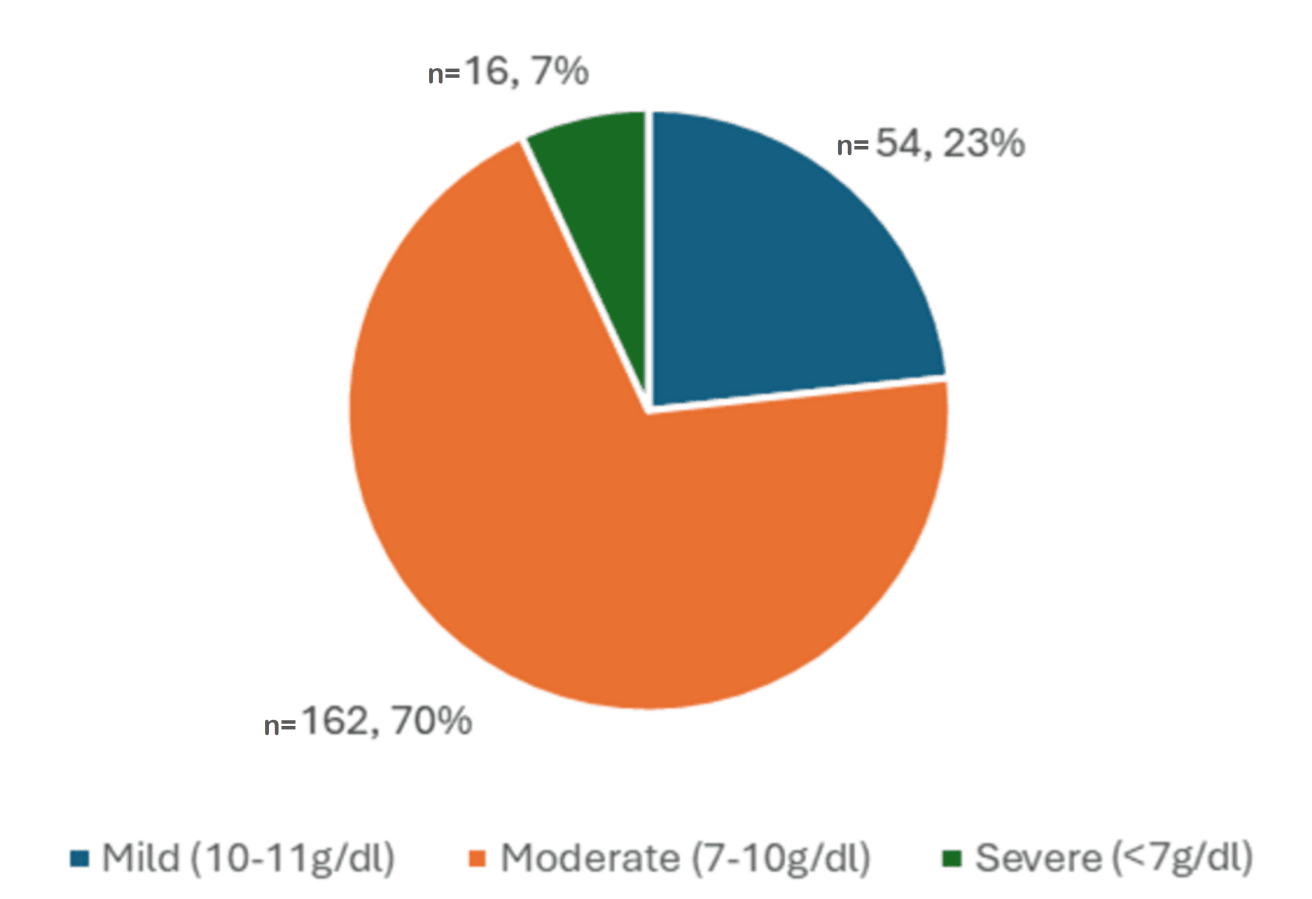
Severity of anemia based on hemoglobin levels in the study population (N=300) The data has been represented as n and %

A total of 232 (77.3%) SAM children were found to be anemic, of which 54 (23.3%) had mild anemia, 162 (69.8%) had moderate anemia, and 16 (6.9%) had severe anemia as depicted in Figure 3 and Table 2.

**Table 2:** Number of children with mild, moderate, and severe anemia The data has been represented as n and %

Severity of Anemia	Hemoglobin range	Number of cases with anemia	Percentage of cases with anemia
Mild	10-10.9 g/dl	162	69.8%
Moderate	7-9.9 g/dl	54	23.3%
Severe	<7 g/dl	16	6.9%

**Figure 4:**
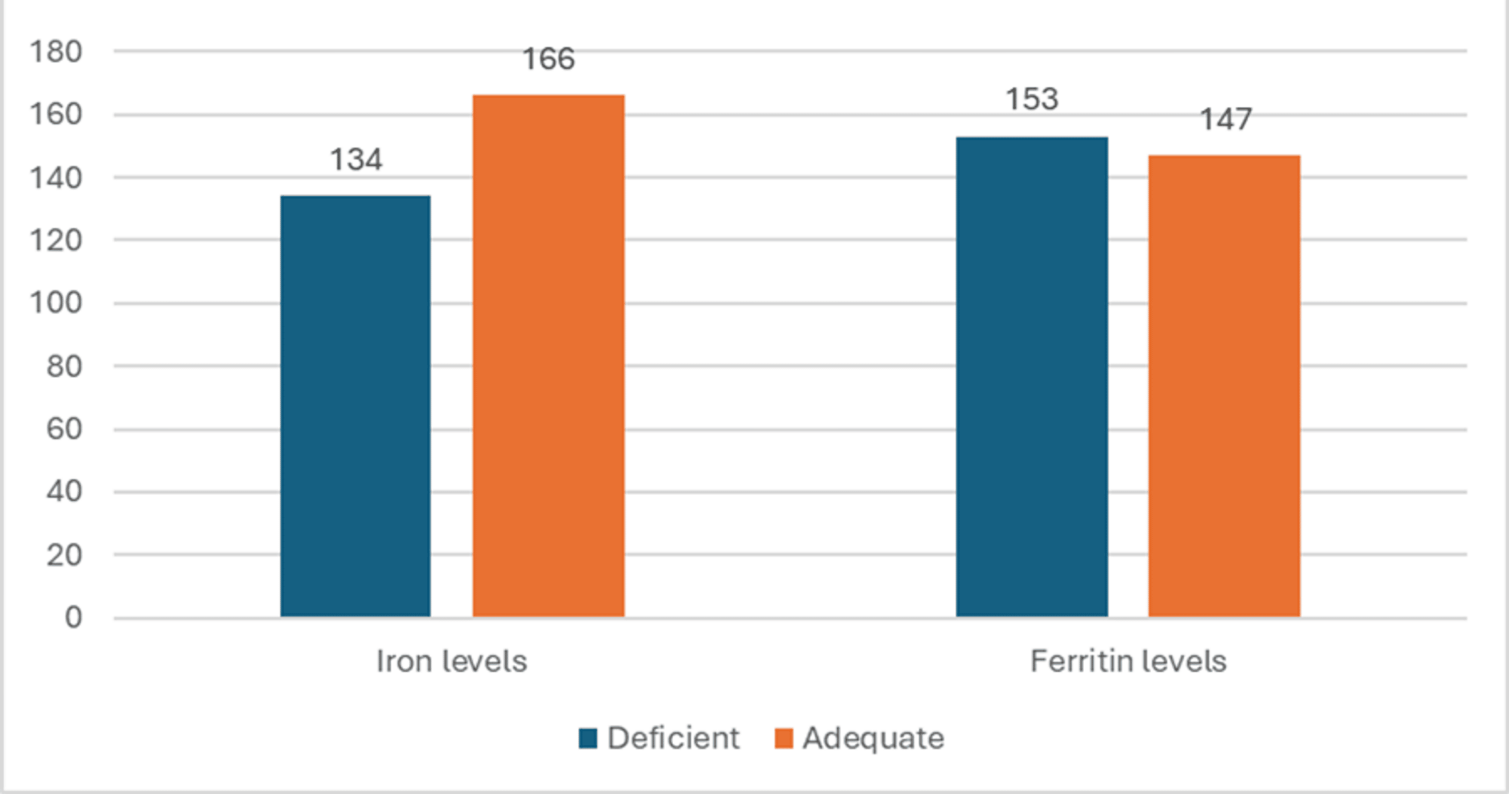
Number of children with deficient and adequate serum levels of iron and ferritin The data has been represented as n

**Figure 5:**
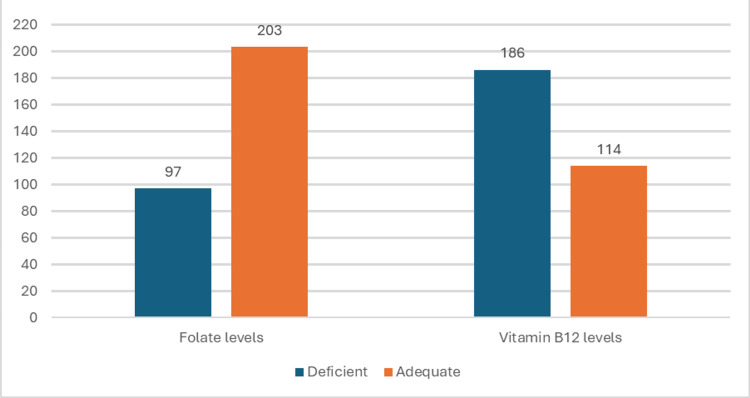
Number of children with deficient and adequate serum levels of folate and vitamin B12 The data has been represented as n

Low serum iron levels were detected in 134 (44.6%) children with iron deficiency being more common in females and below normal ferritin levels were seen in 153 (51%) (Figure 4). Folate levels were found to be deficient in 97 (32.3%) children while vitamin B12 levels were deficient in 186 (62%) (Figure 5, Table 3).

**Table 3:** Number of children with deficiencies of serum iron, ferritin, folate, and vitamin B12 The data has been represented as n, %, and mean

Parameter	Reference Levels	Number of cases with deficiency	Percentage of cases with deficiency	Mean value
Iron levels	50-120 mcg/dL	134	44.6%	128.43
Ferritin levels	50-200 ng/mL for ages 2-5 months, 7-140 ng/mL for ages 6 months-15 years	153	51%	78.43
Folate levels	5-21 ng/mL	97	32.3%	10.17
Vitamin B12 levels	365-1568 ng/L for infants, 345-1154 ng/L for ages 1-3 years, 330-1236 ng/L for ages 4-5 years	186	62%	380.91

**Figure 6:**
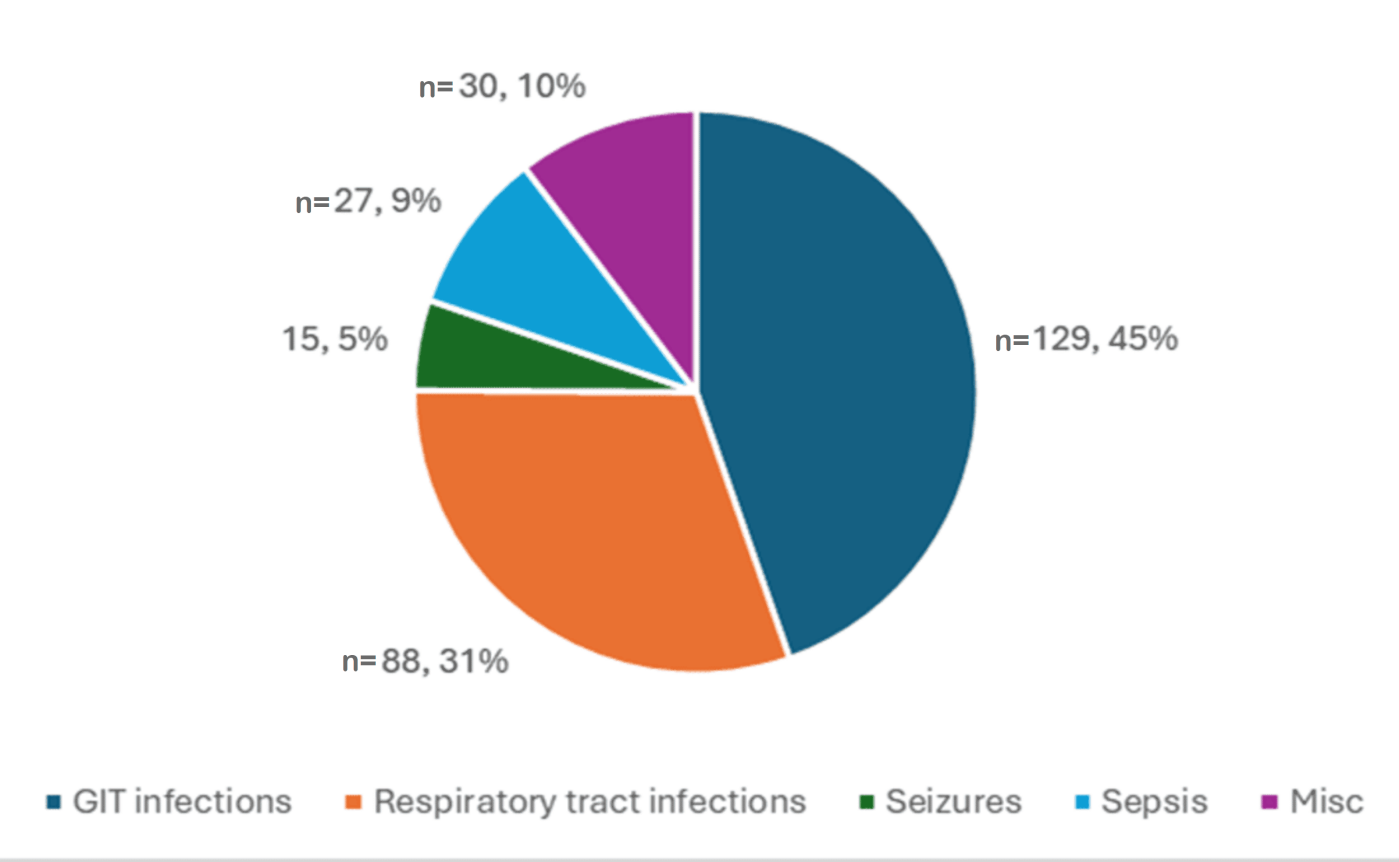
Distribution of various morbidities in the study population (N=300) The data has been represented as n and %

The most common morbidity seen in the children with SAM was gastrointestinal infections presenting as diarrhoea in 129 (43%) followed by respiratory tract infections commonly being bronchopneumonia in 88 (29.3%). Seizures were seen in 15 (5%) children, the causes being metabolic disturbances, febrile seizures, and meningitis. A total of 27 (9%) children presented with sepsis and 11 (3.7%) were diagnosed with dengue. Another 30 (10%) children presented with miscellaneous diseases (urinary tract infections, measles, HIV, skin infections) as depicted in Figure 6.

## Discussion

Children with SAM are prone to infections, and due to nutritional deficiency, many have anemia. Post COVID-19, there has been an increase in malnutrition in children. A study by Ulahannan et al. looks at the changing trends in the prevalence of SAM in various Indian districts across the years. It has increased from 6.6% in 2005-2006 as per the NFHS-3 to 7.5% in 2015-2016 as per the NFHS-4, and further to an alarming 7.7% in 2019-2021, as per the NFHS-5. The prevalence has been on the rise and so has the need for preventive measures [10].

A study by Chandra et al. emphasizes the importance of assessing the severity of anemia and hematopoietic micronutrient deficiencies (folic acid, vitamin b12, iron) in children with SAM. Assessment of these deficiencies and measures taken towards supplementation improve the weight gain and recovery. The study suggested iron supplementation for three to six months to achieve normal hemoglobin levels [11].

Thakur et al., in their study on anemia in children with SAM, noted that of the 131 cases, 81% had anemia of which 67.3% had severe anemia and 13.8% had moderate anemia, the most common type being microcytic (38.6%) followed by macrocytic (30.5%) anemia [1].

A study by Atiq et al. focused on determining the proportion of children with SAM with vitamin B12 deficiency and its effect on their development. In their study, 45 of the total 100 children were vitamin B12 deficient, of which 93% had a gross motor delay, 87% had a fine motor delay, 62% had language delay, and 80% had cognitive-adaptive delays [12].

Shukla et al., in their study, concluded that children with SAM are more likely to suffer from vitamin B12 and iron deficiencies, and severe vitamin b12 deficiency was often associated with severe anemia [13].

In a study by Yaikhomba et al., which assessed the serum parameters (iron, b12, folic acid) in hospitalized SAM children aged 6-59 months, 94% had anemia and there was a strong association between Z-score <-3 SD and vitamin B12 deficiency. The study found that vitamin B12 deficiency was more common than iron and folic acid deficiencies [14].

The current study included 300 children with SAM of which 124 (41.3%) were males and 176 (58.6%) females. The findings were comparable with the study conducted by Sharma et al. [15], in which 60% were females and 40% were males, in contrast with the study conducted by Murthy et al. [16], in which 53.8% were males and 46.2% were females.

Anemia in children is defined as blood hemoglobin <11 g/dl and is classified into mild (10-10.9 g/dl), moderate (7-9.9 g/dl), and severe anemia (<7 g/dl) [17]. The present study observed that 77.3% (n=232) of the cases had anemia, which was comparable with the study conducted by Sharma et al. [15], in which anemia was noted in 73.5% whereas it was to the extent of 95% in the study conducted by Arya et al. [18]. In the current study, 23.2% (n=54) had mild, 69.8% (n=162) had moderate, and 6.89% (n=16) had severe anemia, respectively. The majority of the cases had moderate anemia (69.8%), which was in contrast to the study conducted by Arya et al. [18], in which 52% had severe anemia, followed by moderate anemia (28%), and the study by Thakur et al. [1], in which also, the majority had severe anemia (67.3%) followed by moderate anemia (13.8%) [18,1].

Our study found that serum ferritin levels were deficient in 51% (153) of the cases while iron levels were deficient in only 44.6% (134). This may indicate that a significant part of the study population is in the early stages of iron deficiency anemia where serum ferritin levels, i.e., iron stores of the body were depleted but the serum iron levels were within the normal range. The iron stores may have possibly been depleted in more cases but ferritin being an acute phase reactant is normal or elevated in inflammatory conditions.

In the current study, it was found that vitamin B12 deficiency (62%, n=186) was more prevalent than iron (44.6%, n=134) or folate deficiency (32.3%, n=97). This finding was comparable with the study conducted by Murthy et al. in which they found that 45% of the cases had B12 deficiency and 3.8% had a folate deficiency [16]. In another study conducted by Yahikhomba et al. on the assessment of serum parameters (iron, vitamin B12, and folate) in children with SAM, it was found that vitamin B12 deficiency was more common than folate and iron deficiencies [14]. In a study conducted by Chhabra et al., it was found that nutritional megaloblastic anemia is seen commonly in association with malnutrition in children aged 3-18 months [19]. These results were consistent with our study.

In the present study, the most common morbidities associated with SAM were gastrointestinal infections presenting as diarrhea in 129 (45%) cases followed by respiratory tract infections commonly being bronchopneumonia in 88 (29.3%). These findings were consistent with the study conducted by Sharma et al., which found that the most common morbidities were bronchopneumonia (39.8%) and diarrhea (30.5%) [17]. The study conducted by Kumar et al. concluded that the most common comorbidity in children with SAM was diarrhea (55%) followed by respiratory tract infections (27.8%) [20], the other comorbidities included measles and HIV. These findings were consistent with our study.

The children with SAM were given micronutrients as per the guidelines of the facility-based care of SAM. Folic acid was given at 5 mg on day 1 and then 1 mg/day for two weeks. Iron supplementation on a daily basis was started two days after the child was on a catchup diet. Elemental iron in the dose of 3 mg/kg/day in two divided doses was supplemented for a period of three to six months after discharge. Vitamin B12 supplementation was given in those with a deficiency in the form of oral methylcobalamin 500 mcg/day daily for four to six weeks, then once a week till hematocrit returns to normal.

Recommendations

Measures to prevent anemia and malnutrition in children less than 60 months can be initiated during the antenatal period by way of improvement of maternal nutrition, along with iron and folic acid supplementation. Exclusive breastfeeding in the first six months of age and continuation of breastfeeding along with initiation of home-available complementary feeding from six months of age go a long way in maintaining healthy nutrition in children in the vulnerable age group of less than 60 months. Parents and primary caretakers need to be educated about the infant and young child feeding practices during the well-baby and well-child visits. Growth monitoring during these visits helps in the early identification of growth faltering. These visits ought to be taken as opportunities to identify the presence of malnutrition, and anemia so that corrective steps can be taken for its management which helps prevent severe acute malnutrition and anemia, which is associated with significant morbidity and mortality. 

Limitations

The study has some limitations. The study duration being only two months limited the sample size. The study population had significantly more subjects in the age group of 6-60 months (N=278) as compared to <6 months (N=22), which was a limiting factor. The nutritional status and hemoglobin level of the mothers were not assessed. The feeding practices in the children (breastfeeding, initiation of complementary feeding, and type and frequency of complementary feeds) were not documented in detail in the present study. Future studies should keep these in consideration.

## Conclusions

In the present world, malnutrition on both ends of the spectrum is a concern to be addressed among children. At one end is undernutrition and at the other end is overnutrition, both of which are associated with complications in the short term and long term. Anemia is common in children with SAM with iron deficiency being the commonest. However, as noted in our study, B12 deficiency also needs to be addressed and its supplementation should be included in programs in the management of SAM children.
